# MTHFR Gene Polymorphisms: A Single Gene with Wide-Ranging Clinical Implications—A Review

**DOI:** 10.3390/genes16040441

**Published:** 2025-04-08

**Authors:** Antoni F. Araszkiewicz, Krzysztof Jańczak, Paweł Wójcik, Bartłomiej Białecki, Szymon Kubiak, Michał Szczechowski, Danuta Januszkiewicz-Lewandowska

**Affiliations:** 1 Faculty of Medicine, Poznan University of Medical Sciences, ul. Fredry 10, 61-701 Poznan, Poland; 91167@student.ump.edu.pl (A.F.A.); 91246@student.ump.edu.pl (K.J.); 91470@student.ump.edu.pl (P.W.); 91177@student.ump.edu.pl (B.B.); 91299@student.ump.edu.pl (S.K.); 91435@student.ump.edu.pl (M.S.); 2 Clinic of Oncology, Hematology and Pediatric Transplantology, Poznan University of Medical Sciences, ul. Fredry 10, 61-701 Poznan, Poland

**Keywords:** MTHFR, mutations, polymorphisms, homocysteine, folate, atherosclerosis, cancerogenesis, Alzheimer’s disease, diabetes, NTDs

## Abstract

The enzyme 5,10-methylenetetrahydrofolate reductase (MTHFR) catalyzes the conversion of 5,10-methylenetetrahydrofolate to 5-methyltetrahydrofolate, a process essential for the methylation of homocysteine to methionine. Polymorphisms in the MTHFR gene can reduce enzyme activity, disrupting the folate cycle and leading to hyperhomocysteinemia. The two most common polymorphisms associated with this gene are 667C>T (rs1801133) and 1298A>C (rs1801131). **Background:** This review provides a comprehensive summary of the current knowledge regarding MTHFR polymorphisms, with a particular focus on their potential impact on disease susceptibility. We hope this review will serve as a valuable resource for understanding the significance of MTHFR polymorphisms and their complex relationships with various diseases. **Methods:** For this review, we prioritized recent evidence, focusing on reviews and meta-analyses published between 2015 and 2025, sourced from PubMed and Google Scholar. **Results:** We explore the connection between these polymorphisms and a broad spectrum of medical conditions, including cardiovascular diseases and oxidative stress pathology; neurological and psychiatric disorders, such as Autism Spectrum Disorder, Alzheimer’s disease, Schizophrenia, and Major Depressive Disorder; fertility, pregnancy, and neonatal complications, including recurrent pregnancy loss, pre-eclampsia, preterm birth, low birth weight, and neural tube defects; metabolic disorders, such as diabetes mellitus, inflammatory bowel disease, and non-alcoholic fatty liver disease; and oncological conditions, including breast, prostate, and ovarian cancers; as well as leukemia, and autoimmune diseases, particularly rheumatoid arthritis. **Conclusions:** While some diseases have a well-established association with MTHFR polymorphisms, others require further investigation. Our analysis highlights the crucial role of environmental factors, such as ethnic background and dietary folate intake, in influencing study outcomes.

## 1. Introduction

**Figure 1:**
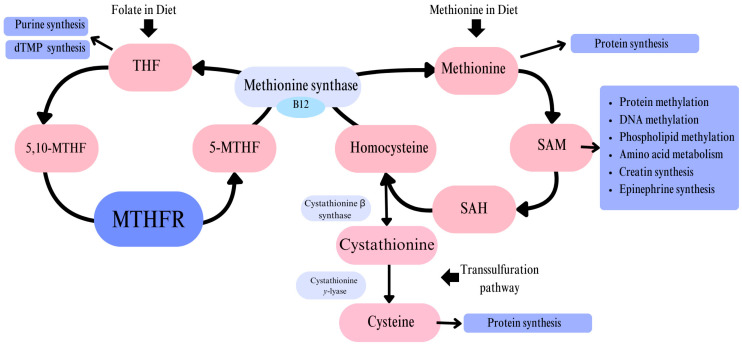
Graphic representation of the reactions of the folate cycle with information specified in the introduction, the destination of the indicated products is shown.

Methylenetetrahydrofolate reductase (MTHFR) is an enzyme responsible for converting 5,10-methylenetetrahydrofolate (5,10-methylene-THF) to 5-methyltetrahydrofolate (5-methyl-THF). MTHFR is a flavoprotein that facilitates NADPH-dependent reduction, utilizing FAD as a cofactor [1]. This conversion is a critical step in the folate cycle [Figure 1], with 5-methyl-THF subsequently serving as a substrate for the methylation of homocysteine (Hcy) to methionine (Met). This reaction is catalyzed by methionine synthase (MS), with vitamin B12 acting as a coenzyme.

The direct products and further derivatives of this reaction serve as one-carbon donors, which are essential for numerous metabolic pathways [2]. These pathways include the synthesis of amino acids, purines, deoxythymidine monophosphate (dTMP), creatine, and epinephrine, as well as DNA and protein methylation [3]. Given the wide range of these processes, MTHFR plays a crucial role in cellular metabolism.

The MTHFR gene is located on chromosome 1p36.22 [4]. It is transcribed alongside other enzymes involved in the folate cycle, such as cystathionine β-synthase (CBS) and methionine synthase (MS), as they share a common promoter region. Alternative splicing has been observed in humans [1]. To date, approximately 247 single-nucleotide polymorphisms (SNPs) [5,6] and over 200 pathogenic variants [6,7] of the MTHFR gene have been identified. Although 34 rare polymorphisms have been linked to severe MTHFR deficiency, research on this topic remains limited, as only around 50 patients have been diagnosed with one of these polymorphisms [8].

Two of the MTHFR gene polymorphisms, 677C>T and 1298A>C, are the most common and widely studied. These are the only two polymorphisms currently known to have a confirmed effect on enzyme activity [8]. The 677C>T (rs1801133) polymorphism is a point mutation that results in the substitution of alanine (Ala) at position 222 with valine (Val) in the enzyme’s catalytic domain (A222V) [1]. This polymorphism increases thermolability [9] and reduces the enzyme’s affinity for FAD, leading to a 35% decrease in enzyme efficiency per mutated allele [1]. The prevalence of this polymorphism in human populations averages 30–40%, but it varies from 0% to over 40%, depending on geographic location, with minimal difference between genders [2]. Homozygous individuals have been diagnosed with mild hyperhomocysteinemia. The 1298A>C (rs1801131) polymorphism is a point polymorphism that causes a substitution of glutamate (Glu) at position 429 with alanine (Ala) (E429A) [9]. Unlike 677C>T, carrying the 1298A>C mutated allele alone does not lead to hyperhomocysteinemia. However, individuals who are 677C>T heterozygotes and also carry the 1298A>C allele exhibit enzyme function similar to 677C>T homozygotes [1]. Both polymorphisms can result in decreased MTHFR activity, potentially disrupting the folate cycle and related metabolic processes.

Hyperhomocysteinemia (HHcy) is a condition characterized by elevated blood levels of homocysteine (Hcy), a non-proteinogenic, sulfur-containing amino acid. It can result from disruptions in the folate cycle, such as MTHFR gene polymorphisms, which impair the methylation of Hcy to methionine (Met). A compensatory defense mechanism exists, in which excess Hcy is converted to cysteine through the transsulfuration pathway [10]. However, this process appears insufficient to fully prevent HHcy.

The study conducted by Nishio et al. suggests that homozygous individuals with the TT genotype (resulting from the 677C>T polymorphism) have 20% lower folate levels compared to individuals without the polymorphism, despite having the same folate intake [11]. This may be particularly significant during periods of high folate demand, such as pregnancy, lactation, or infancy [12]. Individuals with this homozygous polymorphism may require not only folic acid supplementation but also active forms of folate. Single-carbon units, such as tetrahydrofolate (THF) and S-adenosylmethionine (SAM), are derivatives of the folate cycle [3]. These molecules serve as methyl group donors, and their deficiency can lead to DNA, protein, and phospholipid hypomethylation, the degradation of telomere sequences, and disrupted amino acid and nucleotide metabolism [13]. These effects highlight the critical role of folate metabolism in maintaining genetic stability and cellular function.

**Figure 2:**
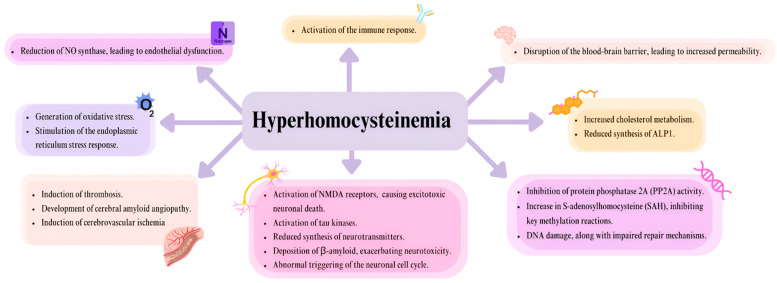
The figure depicts the effects of hyperhomocysteinemia/elevated homocysteine levels on the cardiovascular and the nervous system, focusing on the endothelium and neurons, based on the information provided in the introduction [13,14].

The toxic effects of Hcy, primarily impacting the nervous and circulatory systems, are attributed to multiple mechanisms [Figure 2]. These mechanisms highlight the widespread influence of HHcy on cellular and systemic functions, particularly in the cardiovascular and neurological systems [13,14].

The folate cycle has been extensively researched, particularly in recent years. However, most studies focus either on specific aspects of the metabolic pathway or on the correlation between folate cycle disruptions and particular medical conditions. There appear to be few comprehensive reviews that summarize folate cycle knowledge in a practical, multidisciplinary manner across various medical fields.

It is important to acknowledge the potential for bias in selected research papers utilized in this review. Specifically, the selection of subjects for the research groups may be influenced by factors such as the severity of their condition, the progression of the disease, and their willingness to participate in the study. Similarly, the selection of the control group may also be biased, which could affect the results. The size of some of the groups were limited, which could have an impact on the research’s credibility. This is particularly important because the prevalence of certain diseases and MTHFR polymorphisms varies between ethnic groups. Finally, some of the researchers did not consider the folate intake or dietary habits of the subjects, which could also influence the results.

In this article, we aim to highlight the medical disciplines most relevant to the folate cycle and its associated research. Therefore, the purpose of this review is to examine the most common MTHFR gene polymorphisms, explore their implications across selected medical fields and provide a comprehensive summary of the current knowledge on the topic. By doing so, we hope to provide a broad, yet practical, perspective on the role of MTHFR polymorphisms in health and disease.

## 2. MTHFR in Cardiology

MTHFR polymorphisms and disorders of folate metabolism are the underlying causes of hyperhomocysteinemia (HHcy). The impaired methylation of homocysteine (Hcy) leads to its accumulation in the body, which has been identified as an independent risk factor for cardiovascular diseases. Studies show that up to 40% of patients diagnosed with premature coronary artery disease, vascular disease, or recurrent venous thrombosis have elevated homocysteine levels [15].

Epithelial damage occurs due to Hcy’s tendency to auto-oxidize and the impairment of the vasodilatory response associated with nitric oxide (NO). Hcy also exerts a pro-inflammatory effect on endothelial cells (ECs), inducing the secretion of pro-inflammatory cytokines (IL-1β, IL-6, IL-8, and TNF-α). These cytokines are linked to reactive oxygen species (ROS) formation and the activation of nuclear factor kappa B (NF-κB) [16]. Cytokines recruit monocytes, which differentiate into pro-inflammatory macrophages that transform into foam cells, contributing to plaque formation.

Furthermore, Hcy accelerates endothelial cell (EC) aging by promoting apoptosis and other forms of cell death, including D-gasdermin-dependent pyroptosis [17].

In addition, elevated levels of Hcy impair cellular metabolism by impacting respiratory chain proteins [18]. Studies have shown that homocysteine (Hcy) decreases the activity of complexes II and III in rat myocardial cells decreasing the efficiency of the respiratory chain metabolism [19]. The reactive metabolite of Hcy, thiolactone, can bind to the amino groups of lysine in susceptible proteins of the cell, including the cytochrome c complex, which is involved in the electron transfer from complex III to IV [20]. This results in reduced ATP production and impaired mitochondrial function, which influences cellular aging [21]. It has been shown that Hcy increases superoxide leakage leading to significant ROS production [22]. It has been shown that ROS generation through Hcy auto-oxidation in the presence of copper has been implicated in endothelial cell injury and suggests that Hcy influences Ca^2+^ homeostasis, which is linked to ischemia-reperfusion injury in hypoxic heart cells during myocardial infarction [18,23].

**Figure 3:**
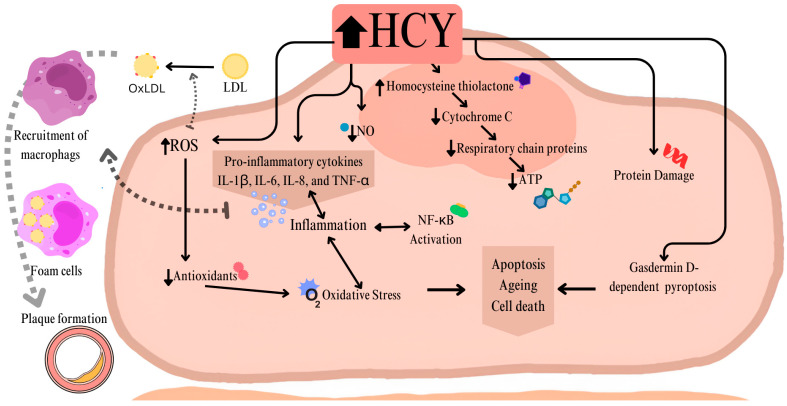
The figure shows the discussed pathomechanism of epithelial damage and its contribution to atherosclerotic plaque formation in hyperhomocysteinemia.

In summary, Hcy affects cell metabolism in various ways including oxidative stress, inhibiting respiratory chain proteins, reducing ATP production, and inducing protein damage. The pro-inflammatory effects on cardiovascular cells contribute to vascular dysfunction and increase the risk of heart disease [Figure 3].

Recent reviews have summarized numerous studies and meta-analyses indicating cardiovascular complications associated with the 677C>T polymorphism, including an increased risk of developing cardiovascular disease (CVD). The 677C>T homozygotes of the MTHFR gene are at a higher risk of developing CVD [24]. However, the same review also included studies that did not find a correlation between the 677C>T variant and CVD in patients with diabetes, though this study had a small sample size of 107 participants [24]. Many studies have shown an increased risk of ischemic stroke (IS) in individuals with the MTHFR gene polymorphism. For example, a study by Moti Wala et al. found an association between MTHFR polymorphism and the dissection of carotid and spinal arteries, which significantly affect the risk of IS at a young age. The review included 11 articles, comprising three case reports and one experimental study with a sample size of 4840 patients [25]. Hyperhomocysteinemia (HHcy) in homozygotes has been shown to be a risk factor for vascular events, including myocardial infarction [26]. Arterial dissection refers to structural damage to the arterial wall with intimal hemorrhage, which forms an intramural hematoma (IMH), resulting in a bulge inside the vessel and the potential for thrombus formation. This condition is also associated with Hcy-related damage mechanisms. IMH hinders perfusion, increasing the risk of embolism [27]. A retrospective study by Ahmed et al., involving 4055 patients, provides evidence of a correlation between vitamin B12 deficiency (present in folate cycle disorders) and ischemic stroke [28]. Another study by Park et al. on 146 patients with thromboembolic disease also found a correlation between thrombosis, HHcy, and the 677C>T polymorphism. The authors concluded that MTHFR gene polymorphism testing could aid in the early diagnosis of arterial dissection, which often cannot be diagnosed based on symptoms like headache or migraine [29].

Polymorphisms that reduce the enzymatic activity of MTHFR cause folate deficiency, which is an independent risk factor for stroke. The correlation between the 677C>T mutation and stroke, due to inconsistent results from studies in different populations, needs further research [30]. The study by Zhao et al. updated previous meta-analyses on this topic by including clinical and cohort studies investigating MTHFR 677C>T polymorphism and stroke susceptibility, focusing on patients diagnosed with stroke for the first time. Non-original studies, duplicate articles, and those where the Hardy–Weinberg equilibrium value was <0.05 were excluded. In total, 96 clinical-cohort studies from different populations and age groups, comprising 314,814 patients, were selected for meta-analysis. In the dominant model, the MTHFR 677C>T polymorphism significantly increased the risk of ischemic stroke (OR = 1.47, 95% CI = 1.33–1.61). A correlation was observed in all age groups except the younger population. In the recessive model, similar results were obtained (OR = 1.52, 95% CI = 1.38–1.81), with a significant increase in risk observed in older individuals. Both heterozygous and homozygous models showed similar results [31]. These findings were consistent with previous analyses [32]. Similarly, a meta-analysis including nine studies (3337 patients) showed a statistically significant increase in the risk of ischemic stroke in Chinese cohorts, with a similar trend observed in other populations [33]. The study by Qin et al. indicates the reduced efficacy of Hcy-lowering treatment in individuals with the 677C>T polymorphism. However, the results should be interpreted with caution due to the lack of prospective studies and the fact that not all study groups were age-specific [30].

## 3. MTHFR in Oncology

The reduced enzymatic activity of methylenetetrahydrofolate reductase (MTHFR) caused by the 677C>T and 1298A>C polymorphisms has been observed to increase the propensity for cancer development. Both polymorphisms are associated with elevated levels of serum homocysteine (Hcy), decreased blood folate levels [34,35], and disturbances in one-carbon metabolism. Both the decrease in S-adenosylmethionine availability, most prominent during folate deficiency [36], and the increase in S-adenosylhomocysteine concentration—a methylation byproduct and inhibitor—have been observed to cause inadequate DNA methylation [37,38,39]. The hypomethylation of critical gene loci may have a carcinogenic effect through the abnormal increase in proto-oncogene expression and increased cell proliferation [40,41]. Moreover, hypomethylated regions of the DNA strands have been shown to demonstrate twofold signs of instability in experimental studies, whether through the raised incidence of deamination to uracil, or through the increased vulnerability to endogenous nucleases [42,43]. Furthermore, disruptions in folate metabolism of this nature have been linked to DNA strand breaks in both animal and human studies [42,44]. Finally, certain studies revealed a depletion of the genome-protective p53 protein mRNA and an escalated rate of strand breaks in the p53 loci caused by folate deficiency; the studies, however, focus on laboratory animals [45,46]. All the aforementioned factors contribute to genomic instability [47]. The reduced folate supply is also linked to impaired nucleotide incorporation and inhibited DNA repair [48,49,50]. These factors, together, make both the 677C>T and 1298A>C polymorphisms particularly relevant to the study of various cancers, including breast, prostate, and ovarian cancers, as well as leukemia [51,52,53,54,55,56,57].

### 3.1. Breast Cancer

A review by Petrone et al. examined 19 studies on the prevalence of MTHFR polymorphisms in breast cancer (BC) across different populations. Although the results were inconclusive, both 677C>T and, to a lesser extent, 1298A>C were found to increase the risk of BC [58]. Notably, the 1298A>C polymorphism was observed to possess a protective role against BC among Kazakh women [59]. Moreover, the 677CT 1298AA diplotype was found to decrease BC susceptibility in the South Asian population [60]. The combined effect of the 677C>T SNP and risk factors, such as alcohol consumption and low folate intake, was also found to increase BC susceptibility [61]. Both polymorphisms were associated with more aggressive cancer biophenotypes (such as HER-2 and TN), and the 1298A>C polymorphism was found to be a risk factor for increased lymph node metastasis, although these studies had sample sizes of less than 300 [62]. These results align with a meta-analysis of 75 studies by Kumar et al., which concluded a moderate overall association between the 677C>T polymorphism and susceptibility to BC. In the Asian population, an increased risk was found across all genetic models. In contrast, in the Caucasian population, a statistically significant association was observed only in the allele contrast model (C vs. T). In the mixed subgroup, significant correlations were observed in the allele contrast, dominant, and co-dominant models. Additionally, the allele contrast model revealed a significantly stronger association between the 677C>T polymorphism and BC in hospital-based studies compared to population-based studies. An analysis of 18 studies with varying results found no overall association between menopausal status and the effect of the 677C>T polymorphism on BC risk [63].

### 3.2. Prostate Cancer

A systematic review by You et al. aimed to clarify the effect of MTHFR polymorphisms on the prevalence of prostate cancer (PC) in men of Asian, Caucasian, and mixed descent. The overall study revealed no significant association between the 677C>T polymorphism and an increased risk of PC. However, an analysis of the Asian subgroup revealed a decrease in PC susceptibility across all five genetic models used [64]. On the other hand, a collective study of 6396 cases and 8913 controls with the 1298A>C polymorphism—while not showing any statistically significant overall increase in susceptibility—revealed an increased risk of PC among American men of mixed descent. The authors speculate that this may be due to region-based risk factors [65,66,67].

### 3.3. Ovarian Cancer

A meta-analysis by Xiong et al. incorporated 16 studies on both MTHFR polymorphisms and their influence on ovarian cancer (OC) susceptibility. No significant overall correlation was established regarding the 677C>T SNP, though it was found to be a risk factor among the Asian subgroups. Since Asians made up only a fraction of the examined participants, the authors speculate that a more balanced distribution of participants might alter the overall results. In terms of the 1298A>C polymorphism, the study (with 6860 participants) differentiated only between the “overall” and “Caucasian” groups, with no significant increase in risk observed in either [68].

### 3.4. Leukemia

Leukemia has not been proven to have a specific cause and is believed to result from a combination of environmental, genetic, and epigenetic factors [47,69,70]. However, multiple studies have suggested that, through disturbances in folate metabolism, MTHFR gene polymorphisms likely negatively affect hematopoietic progenitor cells, triggering leukemia [47,71,72]. Four common types of leukemia have been studied: chronic lymphocytic leukemia (CLL), acute lymphocytic leukemia (ALL), acute myeloblastic leukemia (AML), and chronic myeloblastic leukemia (CML) [73,74].

A meta-analysis by Raoufi et al. included a total of 16 studies on the association of common MTHFR polymorphisms and the risk of CLL. The results failed to show a significant association between the 677C>T variation and CLL in any of the five models used. However, the allelic model (A vs. C) revealed a correlation between increased CLL susceptibility and the 1298A>C polymorphism. It is important to note that, although the individual studies were conducted in different geographical regions, the participants were not grouped based on ethnicity. Moreover, environmental factors and their role in the development of CLL were not considered [73]. A comprehensive meta-analysis by Lien et al. reviewed a total of 97 studies to assess the association between MTHFR polymorphisms and ALL, AML, and CML among participants divided by region and age. A pooled data analysis revealed an overall protective role of the 677C>T CT and TT polymorphisms against the development of leukemia, while the subgroup analysis specified the findings. No significant association was found regarding the prevalence of leukemia in adults. Among American children, the TT genotype decreased the risk of ALL, while the CC and TT genotypes increased it. In children from East Asia and Europe, both mutated variants lowered the risk of leukemia, while the CC wildtype was a risk factor among the latter. On the other hand, a leukemogenic effect was discovered among African and South Asian populations. MTHFR 1298A>C polymorphisms were also found to have a protective role against certain types of leukemia, both overall and in specific subgroups. Among adults and children worldwide, the AC and CC genotypes lowered the risk of CML, while the AA wildtype increased it. Furthermore, both mutated variants displayed a protective effect against ALL in American children, with the CC wildtype showing the opposite effect [74].

Though collectively, the findings of the reviewed studies are generally consistent, a notable discrepancy exists between individual papers, which can be attributed to the specific factors considered, sample sizes, and other conditions. Furthermore, the interaction between environmental factors and MTHFR polymorphisms is only explored in a fraction of the articles. Overall, more comprehensive and precise studies are needed to clarify the complex relationship between the discussed polymorphisms and carcinogenesis in individuals.

## 4. MTHFR in Neurology and Psychiatry

The relationship between MTHFR polymorphisms and neurological and psychiatric disorders is quite complex. HHcy is suspected to cause neurotoxicity [75,76], and most neurological and psychiatric disorders appear to result from a combination of various genetic and environmental factors [77]. MTHFR polymorphisms have been extensively studied in connection with different neurological and psychiatric conditions [78,79]. The aim of this chapter is to summarize some of the findings on the subject.

### 4.1. Autism Spectrum Disorder (ASD)

Autism Spectrum Disorder (ASD) is a group of complex neurodevelopmental disorders characterized by repetitive behaviors, restricted interests, social and communication impairments, sensory hypersensitivity, and difficulty adjusting to changes [77,80,81]. Symptoms related to ASD usually manifest between 12 and 18 months after birth and the diagnosis is typically made at 2 years of age [81]. The male-to-female ratio is thought to be 4:1 [80,82]. The prevalence of ASD is estimated to be around 1% of the child population [77,80,82,83,84]. The etiology of ASD has been widely researched and is currently attributed to a combination of environmental, genetic, and epigenetic factors [77,83,84]. Suggested environmental risk factors include maternal age exceeding 35, chronic hypertension, pre-eclampsia, gestational hypertension, being overweight before or during pregnancy, medication and toxin exposure, socioeconomic factors, and complications during pregnancy and birth [77,80,81]. Studies have shown that monozygotic twins share ASD in about 90% of cases [84], and heritability is estimated at 50% [81]. Over 600 genes have been linked to ASD, with only a few (such as Fragile X, SHANK 3, and CASPR 2) showing a statistically significant connection [77]. The suspected negative influence of MTHFR polymorphisms and HHcy on the nervous system has sparked research into their potential connection to ASD [82]. This is further supported by the fact that epigenetic processes, such as DNA methylation and histone modifications, may also be disrupted by MTHFR polymorphisms [83]. Additionally, it has been noted that high concentrations of methyl donors are required during neurogenesis, and MTHFR polymorphisms may negatively impact their availability [77].

There is some variability in the findings of the analyzed studies. However, there seems to be a trend that the 677C>T polymorphism has a significant link to ASD, while the 1298A>C polymorphism does not [81,82,83]. A systematic review by Roufael et al. suggests that low levels of folic acid and vitamin B12 are associated with ASD [80]. A systematic review by Hoxha et al. adds that scientific focus should be placed on modulating the folate uptake at the blood–brain barrier, via RFC1, folate receptor α (FRα—antibodies against it have been found in the cerebrospinal fluid of ASD children), and proton-coupled folate transporter (PCFT), as a way of managing the effects of MTHFR polymorphisms [77]. Additionally, some studies have investigated supplying folic or folinic acid to ASD children or pregnant mothers. Maternal prenatal supplementation of 600 µg of folic acid a day is believed to reduce the risk of developing ASD in children [77]. Tests involving supplementation for children have shown some improvement in cognitive functions, as cited by Lam et al. [85].

### 4.2. Alzheimer’s Disease

Alzheimer’s disease (AD) is the most common neurodegenerative disorder, accounting for about 60–80% of dementia cases. Those affected suffer from progressive memory decline and worsening cognitive function, which significantly impacts their quality of life [86]. AD is divided into early-onset and late-onset (EOAD/LOAD) variants, with the age of differentiation being 65 years [87]. The etiology of AD is complex and not yet fully understood. Over 50 genes have been linked to AD thus far. Familial EOAD is believed to be caused mainly by polymorphisms in the amyloid precursor protein gene (APP), presenilin1 gene (PSEN1), and presenilin2 gene (PSEN2). Sporadic LOAD is associated with various genetic and environmental factors, with apolipoprotein E (APOE) being the most significant one [87]. HHcy is considered a risk factor for AD, as it affects processes such as DNA methylation and repair, oxidative stress, amyloid β aggregation, tau phosphorylation, vascular endothelial dysfunction, and the secretion of inflammatory mediators like tumor necrosis factor α (TNFα), nuclear factor κB, interleukin 6 (IL-6), and interleukin 8 (IL-8) [86]. Since MTHFR gene polymorphisms may lead to HHcy, several studies have focused on determining the connection between MTHFR polymorphisms and AD, particularly the 677C>T (rs1801133), 1298A>C (rs1801131), and 1793A>G (rs2274976) polymorphisms [87].

A study by Jiang et al., involving 721 AD patients and 365 controls from China, linked the 677C>T and 1298A>C polymorphisms to AD. The 677C>T polymorphism was also associated with an earlier onset, higher Hcy levels, and more severe white matter lesions [87]. Research has also investigated the connection between MTHFR polymorphisms and (amnestic) Mild Cognitive Impairment (aMCI), which is considered a transitional stage between aging and the pathological state of dementia [86,88]. The risk of MCI progressing into AD is estimated at 10–15% per year, compared to 1–2% for unaffected elders [86]. In China and the USA, about 20% of people over 70 years old are affected, with about 40% of them showing potentially reversible changes [88]. A 2021 meta-analysis by Sun et al., involving 8 articles and 2175 patients, did not link the 677C>T polymorphism to MCI susceptibility. However, the authors noted that the small sample size might have affected the results [88]. Another study by You et al., including 60 aMCI patients and 30 control patients, did find a link between the 677C>T polymorphism and structural changes in the patients’ right precunei. Once more, the small sample size may have influenced the results [86].

### 4.3. Schizophrenia

Schizophrenia (SCZ) is a psychiatric condition affecting about 1% of the population. Its symptoms are usually divided into positive and negative. The heritability of SCZ is estimated at about 64–81%, and some studies suggest a strong impact of environmental factors in the development of the condition [89]. Schizophrenia negatively affects the quality of life, and those affected have a higher mortality rate than the general population, largely due to high suicide rates [79].

Attempts to link SCZ to folate and vitamin B12 levels have yielded conflicting results. The correlation between MTHFR polymorphisms and SCZ is also being explored due to the former’s potential neurodegenerative effects. The 677C>T and 1298A>C MTHFR polymorphisms both appeared in the Schizophrenia gene database as part of 24 genetic variants associated with SCZ risk [89]. The reviewed studies indicate some interesting findings. Yoo et al. conducted a meta-analysis of 34 case–control studies and found a link between both mentioned polymorphisms and an increased risk of schizophrenia, without significant differences between age, sex, and ethnicity [89,90]. Some studies, however, only found a significant link between the 677C>T polymorphism and the risk of SCZ [79]. Zhang et al. researched depressive (negative) symptoms of schizophrenia in relation to MTHFR polymorphisms. Their research included 715 patients with stable SCZ. There was no difference in the occurrence of depressive symptoms in patients with and without MTHFR polymorphisms. Interestingly, those with depressive symptoms did have higher average levels of Hcy, which was concluded to be a possible risk factor for SCZ depressive symptoms rather than the MTHFR polymorphisms [76]. Liu et al. tested 957 affected patients and 576 controls and did not find a link between the 677C>T polymorphism and schizophrenia but did find a correlation between the polymorphism and suicidal behavior in SCZ patients. The study, however, was claimed to be limited by the sample size and ethnic inconsistencies [91]. Lam et al. reported a link between the 677C>T polymorphism and the risk and age of onset of schizophrenia. Folate deficiency is reported to aggravate negative symptoms, which is consistent with the results of other studies. Folate treatments for SCZ patients have been tested with inconsistent results [85].

### 4.4. Major Depressive Disorder

Major Depressive Disorder (MDD) is a psychiatric condition characterized by at least two weeks of low mood or loss of interest, which interferes with daily activities [78]. It decreases the overall quality of life, increases healthcare costs, and carries an increased risk of suicide. About 30% of cases are resistant to pharmacological treatment, classifying them as Treatment-Resistant Depression (TRD) [92]. Similarly to other disorders in this chapter, the development of MDD is caused by multiple factors [78].

Several studies have researched the link between MTHFR polymorphisms and depressive disorders, including MDD. A 2022 meta-analysis by Zhang et al. found a link between MDD and the 677C>T MTHFR polymorphism [90]. Halaris et al. mentioned that folic acid and L-methylfolate have been shown to be effective in treating depression, with L-methylfolate being one of the substances licensed by the FDA for this purpose [92]. Asif et al. also speculated that MTHFR polymorphisms might influence migraine with comorbid depression (MID), as both conditions are linked to the 677C>T polymorphism [93].

### 4.5. Other Neurological and Psychiatric Disorders

MTHFR polymorphisms have also been studied in relation to a range of conditions not yet mentioned. A considerable amount of research has connected the 677C>T and, in some cases, the 1298A>C polymorphisms with bipolar disorder [78,79,90]. MTHFR polymorphisms can lead to HHcy, which is considered a risk factor for Parkinson’s disease (PD) and complications associated with its L-DOPA treatment [94]. However, Periñán et al. did not find a direct link between the polymorphisms and PD [75]. ADHD has also been linked to MTHFR polymorphisms, with varying results. Sadeghiyeh et al. and Meng et al. found a connection between ADHD and the 1298A>C polymorphism [79,95]. A study by Aguoulea et al. revealed that women with anorexia nervosa were 2.66 times more likely to carry either the 677C>T or the 1298A>C MTHFR polymorphism compared to unaffected individuals [78,96]. The link between MTHFR polymorphisms and neurological and psychiatric disorders remains uncertain as of the time of writing. However, the topic appears to contain some interesting associations that require further research. There is a lack of broad, multinational studies on this subject, as well as research on MTHFR polymorphisms beyond 677C>T and 1298A>C.

## 5. MTHFR in Gastroenterology and Diabetology

### 5.1. Type 2 Diabetes Mellitus

Diabetes mellitus (DM) is a worldwide disease that affected 10.5% (approximately 537 million) of the global adult population in 2021, according to the International Diabetes Federation. It is estimated that, by 2030, this number will rise to 643 million [97]. DM includes several types, each varying in causes, pathomechanisms, and the onset of symptoms [98]. The most prevalent type is type 2 DM (T2DM), which is diagnosed in 90% of diabetic patients. One of the factors responsible for T2DM is genetic predisposition [99]. T2DM is associated with insulin resistance (IR). HHcy has been suggested to contribute to IR by activating a cascade of reactions that leads to the inhibition of the insulin signaling pathway. In these reactions, activated c-Jun N-terminal kinase is involved in the deactivation of insulin receptor substrate-1 through phosphorylation. HHcy is also connected with oxidative stress through its influence on the production of free radicals and the activity of antioxidative enzymes [1]. Oxidative stress is considered one of the pathomechanisms of T2DM and its complications [100]. β-cells, similarly to the endothelium and neurons, are vulnerable to reactive oxygen species because of their low antioxidant defense efficiency [101]. T2DM causes microvascular and neurological complications in numerous cases. The most common are diabetic nephropathy, diabetic retinopathy, and diabetic neuropathy. It is known that HHcy, which may be caused by MTHFR polymorphisms, is associated with vascular damage [24]. Elevated levels of Hcy, along with the oxidative stress connected to it, are also responsible for disturbances in neuronal function [102].

MTHFR 677C>T and 1298A>C polymorphisms are associated with HHcy [1]. Because of this, these polymorphisms are thought to be risk factors for the development of T2DM through the pathomechanisms. This topic has been explored in several studies. Meng et al. conducted a meta-analysis consisting of 68 studies that found a strong association between the 677C>T polymorphism and T2DM prevalence in the Asian population, but not in Caucasian and African populations [103]. Studies from India and Iran also suggest that this polymorphism may be a risk factor for T2DM [104,105]. However, a recent study from Brazil, performed on 286 patients, suggests that the 677C>T polymorphism is not associated with T2DM [106]. Studies are also inconsistent regarding whether the 1298A>C polymorphism increases the risk of T2DM. Yan et al. observed an association between this polymorphism and an elevated risk of T2DM, especially in the Asian population [107]. However, in a prospective study performed on Chinese adults, the authors concluded that the AC + CC genotypes might have a protective effect against T2DM development [108].

Guan et al. analyzed 28 studies with a total of 4633 Caucasian and 4153 Asian patients to explore the potential association between the 677C>T polymorphism and the risk of diabetic nephropathy (DN). The results suggested the existence of such an association in both ethnic groups [109]. However, Elqadi et al. could not link the 677C>T polymorphism with DN risk, although the study had limited statistical power due to factors such as a small sample size (208 patients) and potential issues with generalizability [110].

Settin et al. conducted research on Egyptian patients and observed that the presence of the 677T allele and the 1298C allele is associated with a susceptibility to diabetic retinopathy (DR) [111]. Although another study found no such association [110], the results of a meta-analysis performed on 18 studies indicate a significant relationship between the 677C>T polymorphism and DR risk in the Asian population [112].

Zhao et al. conducted an extensive meta-analysis on the influence of eight polymorphisms on the occurrence of diabetic peripheral neuropathy (DPN) and found no relationship between the 677C>T polymorphism and the risk of diabetic neuropathy in Asian and Caucasian populations [102]. In contrast, a study with a smaller sample size (90 patients) concluded that such an association existed [113]. However, Zhao et al. suggested that the 1298A>C polymorphism is associated with DPN in Caucasians [102].

### 5.2. Gestational Diabetes Mellitus

Gestational diabetes mellitus (GDM) is a condition of impaired glucose tolerance detected for the first time during pregnancy. Its pathomechanisms include insulin resistance (IR) and the disturbed proliferation of β-cells. In pregnancy, the main cause of IR is elevated levels of placental hormones [114]. Studies conflict regarding whether HHcy is related to GDM risk [115]. Nonetheless, increased Hcy levels might negatively influence IR, and, through this pathway, HHcy could potentially be associated with GDM development [1]. The role of MTHFR polymorphisms, which are responsible for HHcy, has been explored in several studies.

A meta-analysis performed on 17 studies, including 12,345 Chinese patients, shows that the MTHFR 677C>T polymorphism might be positively associated with GDM risk in the southern Chinese population [116]. However, Pham et al., who explored the influence of this polymorphism among Vietnamese pregnant women in their first trimester, concluded that such an association did not exist. However, the sample size of their study was significantly smaller (210 participants) [117]. Dias et al., in their study performed on South African women, also failed to link this polymorphism to GDM, but they found an association between the T allele and higher levels of fasting insulin. The authors suggest that the MTHFR 677C>T polymorphism might participate, among other factors, in the pathomechanisms related to GDM development [118].

### 5.3. Metabolic Syndrome

Metabolic syndrome (MS) is a combination of five pathologies: insulin resistance, central obesity, hyperglycemia, dyslipidemia, and hypertension. HHcy is considered a risk factor for MS through several pathways, such as oxidative stress and cytotoxicity [119]. One of the main pathomechanisms of MS is associated with insulin resistance (IR) [119], and, as mentioned earlier, HHcy contributes to IR [1].

One of the polymorphisms that might be a risk factor for MS development is 677C>T in the MTHFR gene. Azizi et al., in their meta-analysis comprising seven studies conducted between 2002 and 2012, concluded that this polymorphism did not have a significant impact on MS risk [120]. However, a Chinese control study presents conflicting results [121]. The reason for such disagreement may lie in the epigenetics of individuals [120].

### 5.4. Obesity

Obesity is a chronic disease characterized by excessive fat accumulation, which can lead to various disorders, such as type 2 diabetes mellitus (T2DM). HHcy may be associated with the risk of obesity through processes that disrupt lipid metabolism [122]. Hcy could potentially cause these impairments both directly and indirectly, through endoplasmic reticulum (ER) stress or epigenetic mechanisms [122,123].

Fu et al. analyzed 20 studies involving 38,317 patients and found that the MTHFR 677C>T TT genotype is significantly associated with HHcy, and this homozygous gene variant could be a risk factor for obesity development [123]. However, a study from Mexico, involving 316 young participants (average age 19 years), failed to link the 677C>T polymorphism to obesity. Additionally, the authors suggested that this polymorphism might have a protective effect against obesity development, due to the lower cholesterol and triglyceride levels in TT carriers [124].

### 5.5. Non-Alcoholic Fatty Liver Disease

Non-alcoholic fatty liver disease (NAFLD) is a condition characterized by hepatic steatosis, which may progress to non-alcoholic steatohepatitis (NASH) with more severe liver damage [125]. Alcohol consumption is excluded as a cause, as metabolic disturbances primarily drive the pathogenesis of NAFLD. These disturbances include impairments in lipid metabolism pathways that lead to fat accumulation and oxidative stress related to dysfunctional mitochondria [125,126]. Additionally, elevated levels of homocysteine (Hcy) may increase the risk of NAFLD [127]. HHcy, along with epigenetic mechanisms, could be involved in the development of NAFLD [127,128]. Increased Hcy levels contribute to lipid accumulation, endoplasmic reticulum stress, and endothelial damage—processes responsible for liver injury [128]. Insulin resistance (IR), which is also linked to NAFLD, might be a consequence of HHcy [1,128]. One potential cause of HHcy could be MTHFR polymorphisms.

A meta-analysis suggests that the 677C>T polymorphism may be a global risk factor for NAFLD, while the 1298A>C polymorphism is associated with NAFLD risk in Caucasians [129]. Similarly, a study of 4096 Chinese patients indicates an increased risk of the disease in individuals with the MTHFR CT and CC genotypes [130]. However, a study involving 1786 patients from Italy and Finland (European population) found no association between these polymorphisms and NAFLD risk [131]. Wang et al. also suggest that the 677C>T polymorphism is not a risk factor for NAFLD, although it does influence Hcy levels. They propose that whether this polymorphism increases the risk of NAFLD may depend on ethnic, geographical, and individual factors in the populations studied [132].

### 5.6. Inflammatory Bowel Disease

Inflammatory bowel disease (IBD) is a condition characterized by chronic intestinal inflammation, which can be divided into three phenotypes: Crohn’s disease (CD), ulcerative colitis (UC), and IBD—unclassified (U-IBD) [133]. Environmental factors, such as smoking and diet, may influence the risk of CD and UC [134]. IBD can also have polygenic, monogenic, or pharmacogenetic origins. Monogenic IBD is more common in children than in adults. However, in a gene panel consisting of 75 genes responsible for monogenic IBD, MTHFR was not included [135]. Despite this, polymorphisms, including those in the MTHFR gene, may be associated with IBD in the adult population, and this relationship has been explored in numerous studies. HHcy, potentially caused by MTHFR polymorphisms, triggers the increased expression of molecules involved in inflammatory processes and the hypomethylation of proinflammatory genes, resulting in their activation. Elevated Hcy also disrupts cellular antioxidant defense and may be associated with neoangiogenesis in the intestinal mucosa [136]. The results of studies investigating the relationship between MTHFR polymorphisms and IBD are inconsistent. Varzari et al. concluded that the 677C>T and 1298A>C polymorphisms were not associated with the risk of UC in the Moldavian population. However, they found that the 677 TT genotype might be associated with a less severe course of UC. These findings conflicted with previous studies. The authors also found that the 1298AC genotype, but not the 1298CC genotype, was correlated with a more severe subtype of UC [137]. According to Karban et al., among the Non-Ashkenazi Jewish population, the 677C>T polymorphism was found to increase the risk of CD, but not UC [138].

## 6. MTHFR in Pregnancy and Neonatology

### 6.1. Infertility

Correct cell division during spermatogenesis and oogenesis requires THF for dTMP synthesis [139], while genomic imprinting depends on SAM and DNA methyltransferases (DNMTs) to create a parent-specific DNA methylation pattern [140]. These epigenetic marks influence zygotic development and fetal growth, and are maintained throughout adult life. Furthermore, histone tail modification and chromatin remodeling are also dependent on folate cycle derivatives. Therefore, MTHFR polymorphisms may contribute to infertility [140]. More et al. examined 127 infertile Indian men, of whom 9.7% were 677C>T mutants, compared to only 1.6% in the fertile control group [141]. Another study, conducted by Ménézo et al., shows that the prevalence of 677C>T and 1298A>C homozygous or compound heterozygous polymorphisms reaches 40% in infertility consultation patients [142]. These patients also have higher Hcy levels, which, according to the authors, increase oxidative stress and methylation errors during gametogenesis [142,143]. On the other hand, Ren et al. demonstrated a weak association between MTHFR polymorphisms and semen abnormalities in a study group of 1049 men [144]. A meta-analysis by Liu et al. suggests an association between the 677C>T polymorphism and male infertility, but with inconclusive results in European and African populations [143]. Gong et al. show a correlation between increased semen abnormalities and the 677C>T polymorphism in Asians and Europeans [145]. Notably, previous meta-analyses have been inconsistent, and no further meta-analyses have been published in the last 5 years. Further studies are needed to assess whether the findings depend on ethnic background and dietary folate intake, as is strongly suggested.

### 6.2. Recurrent Pregnancy Loss

HHcy, which frequently occurs in patients with MTHFR polymorphisms [1], is a risk factor for several pregnancy complications [115]. Notably, Hcy levels are lower during pregnancy than in non-pregnant women due to an increase in the glomerular filtration rate, plasma volume, and enhanced fetal absorption [146]. Recurrent pregnancy loss (RPL), defined as two or more early miscarriages, can be caused by HHcy, among other factors [115,147]. Hcy induces vascular inflammation and damages placental and uterine blood vessels, leading to thrombosis and subsequent miscarriage [147]. Placental abruption and the resulting fetal hypoxia are often the causes of a miscarriage [146]. A prospective study by Basha et al. found that the prevalence of 677C>T homozygous polymorphisms was significantly higher in women with RPL than in controls (10% vs. 2.5%). The 1298A>C polymorphism was also more common in the treatment group (13.3% vs. 5%) [148]. The same association was described by Mehta et al. in a meta-analysis that included 5888 cases and 8401 controls. Interestingly, the 1298A>C polymorphism was identified as a risk factor for RPL in Caucasians but not in Asians [149]. However, some previous studies have found the association between MTHFR polymorphisms and RPL to be insignificant [150,151].

### 6.3. Pre-Eclampsia

Endothelial inflammation occurring in HHcy is a potential cause of pre-eclampsia (PE), a condition with a complex etiology [115,152,153]. It is unclear whether HHcy is a consequence of MTHFR polymorphisms [154], as it can also be associated with hypertension, folate deficiency, drug use, impaired renal function, alcoholism, and smoking [153]. Zhang et al. reported a significant association between HHcy and PE, but MTHFR polymorphisms did not appear to increase Hcy levels [155]. Previous studies have been inconclusive, and few studies have been conducted in the last 10 years [156].

### 6.4. Preterm Birth and Low Birth Weight

Preterm birth (PTB), defined as delivery before 37 weeks of gestation, may be associated with HHcy and MTHFR polymorphisms [115]. Abnormal placental vasculature and increased hormone secretion in HHcy may accelerate delivery [115,146]. Elevated Hcy levels in preterm newborns seem to support this theory [157]. A study by Huang et al. involving 1995 pregnant Chinese women revealed a higher prevalence of PTB in mothers who were homozygous for the 677C>T polymorphism. The authors consider hypertensive disorders and gestational diabetes mellitus as potential causes of these PTBs [158], both of which may be caused by HHcy and pose a threat to pregnancy [146]. On the other hand, Wang et al. found MTHFR polymorphisms to be insignificant in the incidence of PTB [159]. Research by Dević Pavlić et al. found no relevant DNA methylation disruptions in MTHFR mutants, although the influence of HHcy was not studied [160]. A meta-analysis conducted by Wu et al. associates PTB with the maternal 677C>T MTHFR polymorphism, particularly in homozygous cases. Notably, a similar association with neonatal polymorphisms has not been established [161]. Some authors suggest that maternal Hcy levels may serve as a potential marker for preterm delivery [157].

Low birth weight (LBW) in newborns may also be associated with MTHFR polymorphisms in the infant or mother, although conclusive evidence has not yet been found. The influence of Hcy on LBW, however, is clearer [146]. Wu et al. found no association between these polymorphisms and LBW [161]. On the other hand, Dipitika et al. described the birth weights of full-term and preterm infants, noting a significant difference between those born to mothers with and without the 677C>T polymorphism [162].

### 6.5. Neural Tube Defects

The development and closure of the neural tube require derivatives from the folate cycle [163,164]. Mothers with MTHFR polymorphisms are likely to have reduced folate levels, which may result in their children developing neural tube defects (NTDs), such as anencephaly or spina bifida. NTDs can lead to fetal death or lifelong disability [165]. It is also suspected that DNA damage repair systems depend on the folate cycle, and their failure may contribute to the occurrence of NTDs [164]. A meta-analysis of 42 studies by Almekkawi et al. confirms a strong correlation between the MTHFR 677C>T polymorphism and the incidence of NTDs [166]. Furthermore, Li et al. show an association between both maternal and fetal 677C>T polymorphisms and NTDs [167]. On the other hand, a meta-analysis by Soleimani-Jadidi et al. found no association between the maternal MTHFR 1298A>C polymorphism and any type of NTDs [168].

The neural tube closes during the third and fourth weeks of gestation. Therefore, to prevent NTDs, it is important to start folic acid supplementation (400 μg/day) when attempting conception [165]. Folic acid has been successfully used for NTD prevention. However, supplementation with 5-MTHF (the active form of folate) instead of folic acid bypasses the MTHFR block, which is crucial for patients with MTHFR polymorphisms [139]. High-dose folic acid supplementation is also a risk factor for the development of unmetabolized folic acid (UMFA) syndrome, which involves the accumulation of excess folate that could potentially have an oxidizing effect [169,170]. We found that 5-MTHF supplementation is also less likely to cause hematological symptoms associated with vitamin B12 deficiency [170].

In conclusion, there is evidence suggesting that 5-MTHF supplementation may replace folic acid, particularly in patients with MTHFR polymorphisms. However, further research is needed on this topic [170,171].

## 7. MTHFR in Rheumatoid Arthritis

Rheumatoid arthritis (RA) is an autoimmune disease characterized by synovitis, which leads to damage to joint cartilage and bone. The etiology of the disease is not precisely defined, but it is generally agreed that both environmental and genetic factors can influence its onset and progression. The disease is more common in women (especially after the age of 65), with a female-to-male ratio of 3:1 [172]. Regarding the genetic factors that affect the risk of RA, MTHFR polymorphisms 677C>T and 1298A>C have been implicated, with their prevalence varying by population. The presence of the 677C>T polymorphism is estimated at 34.1% in Europe, 19.7% in Asia, and 10.3% in Africa [173]. For the 1298A>C polymorphism, the prevalence in Caucasians is 34%, while, in Africans, it is 9% [174]. A consensus has been reached regarding the impact of the MTHFR 1298A>C polymorphism on an increased risk of RA in the general population. However, the association between the 677C>T polymorphism and RA is inconsistent. The impact of MTHFR polymorphisms was addressed in a meta-analysis by Cen et al., who concluded that the MTHFR 677C>T polymorphism increases genetic susceptibility to RA in Asians, while the MTHFR 1298A>C polymorphism increases susceptibility in the general population [175].

Another meta-analysis by Bagheri-Hosseinabadi et al. found that the MTHFR 677C>T polymorphism is associated with a higher risk of RA in the African population, but no association was found in Caucasians and Asians. However, it should be noted that, due to the number of studies available for analysis, relative to ethnicity, the results may not be fully conclusive. In the case of MTHFR 1298A>C, an association with a higher susceptibility to RA in the general population was observed. However, the same association was not found in each racial group [176]. Yi et al. indicated that the MTHFR 677C>T and 1298A>C polymorphisms result in a higher susceptibility to RA in the general population. When broken down into subgroups, they noted an increased risk of RA in Caucasians and Africans, but not in Asians with the 677C>T polymorphism. With the 1298A>C polymorphism, a subgroup analysis showed no significant association with RA in Caucasians and Africans [177].

## 8. Conclusions

**Figure 4:**
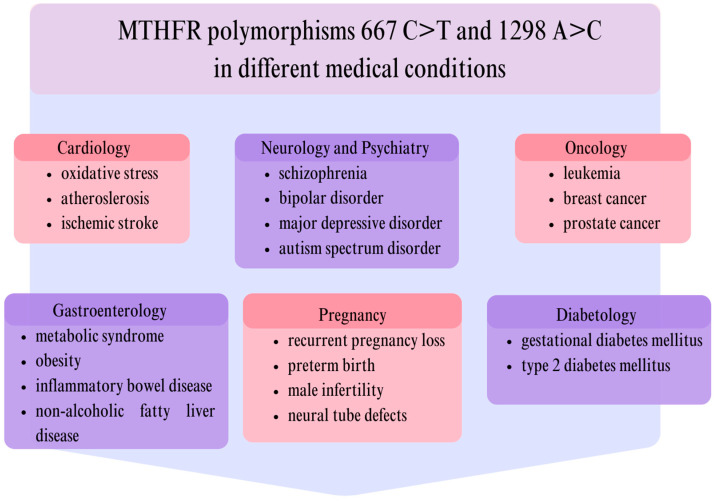
Selection of diseases with a possible connection to MTHFR 677C>T and 1298A>C polymorphisms. Here are some of the most important articles on the subject of MTHFR and the following associated issues: oxidative stress—Kaplan et al. [18]; atherosclerosis—Yuan et al. [16], and Raghubeer and Matsha [24]; ischemic stroke—Ahmed et al. [28], Qin et al. [30], Zhao et al. [31], and Song et al. [32]; schizophrenia—Meng et al. [79], Yoo et al. [89], and Zhang et al. [90]; bipolar disorder—Khan et al. [78], Meng et al. [79], and Zhang et al. [90]; major depressive disorder—Zhang et al. [90], and Asif et al. [93]; autism spectrum disorder—Qiu et al. [81], Li et al. [82], and Razi et al. [83]; leukemia—Raoufi et al. [73], and Lien et al. [74]; breast cancer—Petrone et al. [58], and Kumar et al. [63]; prostate cancer—You et al. [64]; metabolic syndrome—Wang et al. [121]; obesity—Fu et al. [123]; inflammatory bowel disease—Varzari et al. [137], and Karban et al. [138]; non-alcoholic fatty liver disease—Sun et al. [129]; male infertility—Gong et al. [145]; preterm birth—Huang et al. [158], and Wu et al. [161]; recurrent pregnancy loss—Mehta et al. [149]; neural tube defects—Almekkawi et al. [166], and Li et al. [167]; gestational diabetes mellitus—Tan and Chen [116]; type 2 diabetes mellitus—Meng et al. [103], and Yan et al. [107].

In this review, we have summarized the current state of knowledge on MTHFR polymorphisms, and the diseases associated with them. Some of these conditions have been clearly linked to MTHFR polymorphisms, while others require further research. The following figure illustrates the conditions that have been demonstrated to be more clearly linked to MTHFR polymorphisms. In addition, the description of the figure provides a list of some of the most significant papers in their respective areas of research [Figure 4].

It is important to recognize that ethnic background and a traditional diet rich in folate have a significant impact on the results of current studies. In many regions, additional prospective studies are necessary in order to determine whether MTHFR polymorphisms influence disease development and treatment effectiveness. It remains unclear whether routine genetic testing for these polymorphisms is necessary or whether current treatment regimens should be adjusted accordingly. Specifically, such recommendations may, in the future, become standard procedures for diagnosing cardiovascular diseases and folate supplementation during pregnancy. In recent years, there has been a growing number of studies on MTHFR polymorphisms, with new publications emerging annually. To ensure the effective organization of future research, it is crucial that we thoroughly verify new hypotheses.

## Data Availability

The authors of the manuscript hereby formally declare that the research presented herein is based primarily upon publicly accessible scientific sources and scholarly materials acquired through institutional university subscriptions. Furthermore, should any challenges or ambiguities arise regarding access to this manuscript or the source materials referenced within, the authors commit to providing the full text of the work and offering comprehensive assistance to ensure the transparency and accessibility of the research. This declaration is made in adherence to principles of scholarly integrity and with a commitment to facilitating the dissemination and verification of scientific knowledge.

## Abbreviations

The following abbreviations are used in this manuscript:

MDPI	Multidisciplinary Digital Publishing Institute
MTHFR	5,10-methylenetetrahydrofolate reductase; Methylenetetrahydrofolate reductase
NTDs	Neural tube defects
5,10-methylene-THF	5,10-methylenetetrahydrofolate
5-methyl-THF	5-methyltetrahydrofolate
NADPH	Nicotinamide adenine dinucleotide phosphate
FAD	Flavin adenine dinucleotide
Hcy	Homocysteine
Met	Methionine
MS	Methionine synthase
THF	Tetrahydrofolate
5,10-MTHF	5,10-methylenetetrahydrofolate
5-MTHF	5-methyltetrahydrofolate
SAM	S-Adenosyl-L-methionine
SAH	S-Adenosyl-L-homocysteine
dTMP	Deoxythymidine monophosphate
DNA	Deoxyribonucleic acid
CBS	Cystathionine β-synthase
SNPs	Single nucleotide polymorphisms
Ala	Alanine
Val	Valine
Glu	Glutamine
HHcy	Hyperhomocysteinemia
A	Adenine
C	Cytosine
T	Thymine
G	Guanine
NO	Nitric Oxide
NMDA	N-methyl-D-aspartate
PP2A	Protein phosphatase 2A
ALP1	Apolipoprotein 1
EC	Endothelial cells
IL-1β	Interleukin 1 β
IL-6	Interleukin 6
IL-8	Interleukin 8
TNF-α	Tumor necrosis factor
ROS	Reactive oxygen species
NF-κB	Nuclear factor kappa B
CVD	Cardiovascular disease
IMH	Intramural hematoma
OR	Odds Ratio
CI	Confidence Interval
BC	Breast cancer
HER-2	Receptor tyrosine-protein kinase erbB-2
PC	Prostate cancer
OC	Ovarian cancer
CLL	Chronic lymphocytic leukemia
ALL	Acute lymphocytic leukemia
AML	Acute myeloblastic leukemia
CML	Chronic myeloblastic leukemia
ASD	Autism Spectrum Disorder
Fragile X	Fragile X Messenger Ribonucleoprotein 1
SHANK 3	SH3 and multiple ankyrin repeat domains 3
CASPR 2	Contactin-associated protein-like 2
RFC1	Replication factor C subunit 1
FRα	Folate receptor α
PCFT	Proton-coupled folate transporter
AD	Alzheimer’s disease
EOAD	Early-onset Alzheimer’s disease
LOAD	Late-onset Alzheimer’s disease
APP	Amyloid precursor protein
PSEN1	Presenilin 1
PSEN2	Presenilin 2
APOE	Apolipoprotein E
aMCI	amnestic Mild Cognitive Impairment
SCZ	Schizophrenia
MDD	Major Depressive Disorder
TRD	Treatment-Resistant Depression
FDA	The United States Food and Drug Administration
MID	Migraine with comorbid depression
PD	Parkinson’s disease
L-DOPA	L-3,4-dihydroxyphenylalanine
ADHD	Attention deficit hyperactivity disorder
DM	Diabetes mellitus
T2DM	Type 2 diabetes mellitus
IR	Insulin resistance
DN	Diabetic nephropathy
DR	Diabetic retinopathy
DPN	Diabetic peripheral neuropathy
GDM	Gestational diabetes mellitus
MS	Metabolic syndrome
ER	Endoplasmic reticulum
NAFLD	Non-alcoholic fatty liver disease
NASH	Non-alcoholic steatohepatitis
IBD	Inflammatory bowel disease
CD	Crohn’s disease
UC	Ulcerative colitis
U-IBD	Unclassified inflammatory bowel disease
DNMTs	DNA methyltransferases
RPL	Recurrent pregnancy loss
PE	Pre-eclampsia
PTB	Preterm Birth
LBW	Low birth weight
UMFA	Unmetabolized folic acid
RA	Rheumatoid arthritis
